# Methylation of DNA duplexes regulates cGAS-mediated innate immune activation *via* condensate formation

**DOI:** 10.1039/d6cb00059b

**Published:** 2026-06-04

**Authors:** Kunihiko Morihiro, Manami Baba, Moeko Yamada, Akimitsu Okamoto

**Affiliations:** a Department of Chemistry and Biotechnology, Graduate School of Engineering, The University of Tokyo, 7-3-1 Hongo Bunkyo-ku Tokyo 113-8656 Japan morihiro@chembio.t.u-tokyo.ac.jp okamoto@chembio.t.u-tokyo.ac.jp

## Abstract

Exogenous DNA activates innate immunity *via* the cGAS–STING pathway, but the impact of chemical modifications has not been investigated sufficiently. Here, we show that DNA duplexes containing 2′-O-methyl ribonucleotides enhance cGAS-driven liquid–liquid phase separation and enzymatic activity, function effectively in DNA nanotherapeutics in living cells, and enable the rational control of innate immune signaling.

## Abstract 2

Systematic comparison of DNA chemical modifications identifies 2′-OMe-modification as a potent enhancer of cGAS-mediated condensate formation and innate immune signaling.

## Graphical abstract

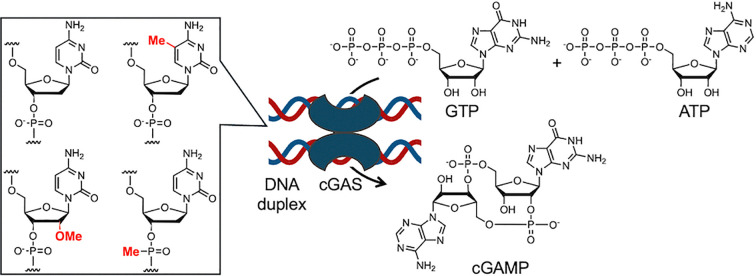

The immune system is a defense mechanism that protects organisms from pathogens such as viruses and bacteria, and is broadly categorized into innate and adaptive immunity. Innate immunity plays a critical role in the early phase of host defense by rapidly sensing invading foreign substances and initiating inflammatory responses. Pathogens possess distinct molecular structures known as pathogen-associated molecular patterns, which are recognized by pattern-recognition receptors (PRRs) expressed in host cells, thereby triggering intracellular signaling cascades. Well-characterized PRRs include Toll-like receptors (TLRs), such as TLR9, which recognizes CpG DNA; RIG-I-like receptors, including RIG-I and MDA5, which detect viral RNA; NOD-like receptors; and C-type lectin receptors.^1^ More recently, a new class of PRRs, termed cyclic GMP–AMP synthase-like receptors, has been identified. Among them, cyclic GMP–AMP synthase (cGAS) functions as a cytosolic sensor for double-stranded DNA (dsDNA), leading to the induction of type I interferons.^2^ Under normal conditions, DNA is confined to the nucleus. However, during infection by viruses or other pathogens, foreign dsDNA can appear in the cytoplasm. Upon recognizing and binding to long dsDNA, cGAS becomes activated and catalyzes the synthesis of the second messenger cyclic GMP–AMP (cGAMP) from ATP and GTP (Fig. 1A). cGAMP then binds to STING, a transmembrane protein localized to the endoplasmic reticulum, inducing its translocation to the Golgi apparatus. During this process, STING recruits and activates the kinase TBK1 *via* its C-terminal domain. Activated TBK1 phosphorylates the transcription factor IRF3, which then translocates into the nucleus to induce the expression of type I interferons, ultimately leading to apoptosis.^3^

**Fig. 1:**
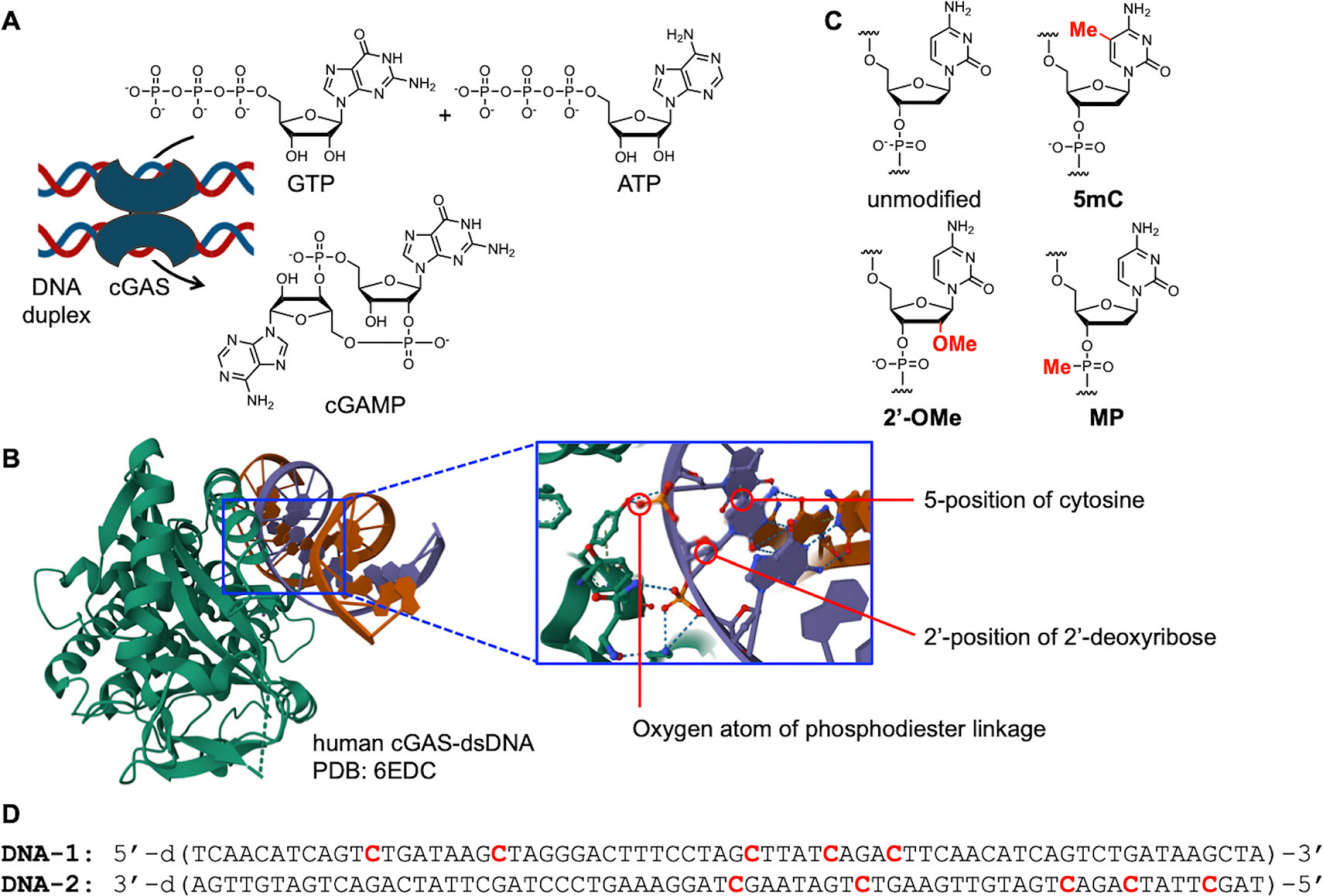
Chemical modifications of DNA duplexes for regulating cGAS activity. (A) Schematic of cGAMP production by cGAS upon activation by a DNA duplex. (B) Interaction between human cGAS and a 16-bp DNA duplex. (C) Chemically modified nucleotides examined in this study. (D) Sequences of the DNA strands used in this study. Chemically modified nucleotides are highlighted in red.

Recognition of dsDNA by cGAS is sequence-independent, and the length of the DNA duplex is considered the most representative factor influencing cGAS activation. DNA duplexes shorter than 45 base pairs fail to activate cGAS, whereas duplexes longer than this threshold proportionally enhance cGAS activity as the length increases.^4^ Two key mechanisms have been proposed to explain the length-dependent activation of cGAS. First, cGAS dimerization is essential. cGAS contains two positively charged DNA-binding grooves, termed Sites A and B, which interact electrostatically with the negatively charged phosphate backbone of DNA.^5^ For full activation, both Sites A and B must bind to DNA to form a cGAS dimer–dsDNA complex, which induces a conformational change in the catalytic pocket necessary for ATP and GTP binding.^6^ Longer DNA duplexes facilitate this process by allowing two cGAS molecules to simultaneously bind adjacent sites along the same DNA molecule in a ladder-like arrangement, promoting dimerization and enhancing enzymatic activity (Fig. S1).^4^ The second proposed mechanism is the formation of biomolecular condensates through liquid–liquid phase separation (LLPS), which is a physicochemical phenomenon in which two aqueous solutions with different solute concentrations separate into distinct phases, much like oil and water. Although this behavior has long been known in physical chemistry, its crucial role in intracellular organization has only recently been appreciated.^7^ Within cells, LLPS can lead to the formation of liquid-like condensates enriched in proteins and nucleic acids, thereby promoting the efficiency of various biological reactions.^8^ Recent studies have reported that cGAS also undergoes LLPS during its activation process, forming liquid-like condensates upon complexation with DNA duplexes.^9^ These condensates are mainly driven by multivalent interactions between cGAS and DNA. Because longer DNA duplexes possess more negatively charged phosphate groups, they more readily induce condensate formation and thereby enhance cGAS activity. Although considerable progress has been made in understanding the relationship between DNA duplex length and cGAS activation, the roles of other factors remain largely unexplored.

Chemical modifications of DNA and RNA molecules are essential strategies for enhancing their practicality as nucleic acid therapeutics. Modification sites on nucleic acids are typically categorized into three regions: the nucleobase, the sugar moiety, and the phosphodiester backbone. To date, numerous chemically modified nucleic acids have been developed.^10^ These modifications improve nuclease resistance, target-binding affinity, and pharmacokinetic properties of nucleic acid drug candidates. However, the impact of these chemical modifications on innate immune activation, particularly through the cGAS–STING pathway, remains insufficiently explored. Based on the crystal structure of the complex formed between human cGAS and a 16-bp DNA duplex,^11^ cGAS is shown to directly recognize oxygen atoms through positively charged amino acid residues (Fig. 1B). This observation suggests that chemical modifications of the phosphodiester linkages in DNA duplexes could substantially affect their ability to activate cGAS. Although nucleobase and sugar modifications of DNA duplexes do not directly participate in the canonical cGAS–DNA binding interface, they can significantly influence the overall interaction landscape by modulating the physicochemical and thermodynamic environment. For example, nucleobase modifications (*e.g.*, at the 5-position of cytosine) can alter the hydration pattern of the major groove, whereas sugar modifications (*e.g.*, at the 2′-position of the furanose ring) can affect DNA duplex conformation, thereby modulating cGAS interaction and activation.

Here, we focused on three types of chemical modifications to DNA duplexes, 5-methylcytosine (5mC) as a nucleobase modification, 2′-*O*-methyl RNA (2′-OMe) as a sugar modification, and methyl phosphonate (MP) as a phosphodiester backbone modification, to investigate the impact of methyl group addition at each site (Fig. 1C). The 5mC modification is a well-known epigenetic marker involved in the regulation of gene expression. TLR9, a pattern-recognition receptor that recognizes CpG motifs, distinguishes mammalian DNA from bacterial and viral DNA based on their methylation status; methylated DNA exhibits reduced immunostimulatory activity mediated by TLR9.^12^ Because the methyl group of 5mC is positioned in the major groove of the DNA duplex, it could potentially alter the binding affinity of cGAS. The 2′-OMe modification induces a more A-form–like structure in DNA duplexes, as the sugar adopts a 3′-*endo* conformation, which is typical of RNA/RNA and DNA/RNA duplexes. This structural shift from B-form to A-form may influence the recognition of DNA by cGAS. 2′-OMe–modified RNA also functions as an antagonist of TLR7, a pattern-recognition receptor that recognizes single-stranded RNA and induces the production of interferons and cytokines.^13^ By contrast, MP directly modifies the phosphodiester backbone, which is known to be a key recognition site for cGAS,^14^ and thus is expected to affect the DNA–cGAS interaction directly. Our chemical approach is promising not only for elucidating the mechanism of DNA duplex recognition by cGAS but also for developing more effective vaccine adjuvants and immunotherapeutics.

We hypothesized that modifying the structure and charge of dsDNA could alter its binding affinity for cGAS and its propensity to form phase-separated condensates, thereby enabling control over cGAS activity. To test this hypothesis, we prepared two complementary 68-mer single-stranded DNA (ssDNAs), designated DNA-1 and DNA-2, and introduced three types of chemical modifications at five cytosine residues on each strand (Fig. 1C). The DNA sequences were designed based on our previous work, which reported a hairpin DNA pair that selectively activates the cGAS–STING pathway in cancer cells.^15^ The resulting dsDNA exceeded the reported activation threshold for cGAS (45 bp). Owing to the synthetic difficulty of modified DNA, especially MP-containing DNA, each strand was modified at no more than five sites. To ensure balanced modification of the dsDNA, the modified nucleotides were placed at relatively 5′-proximal positions in DNA-1 and DNA-2 (Fig. 1D).

The dsDNAs with each chemical modification were prepared by annealing two complementary ssDNAs, DNA-1 and DNA-2. To confirm duplex formation, 2 µM each of DNA-1 and DNA-2 were annealed in a buffer containing 50 mM NaCl, TE buffer, and 1.25 mM MgCl_2_, followed by analysis using native PAGE. In the gel image (Fig. 2A), the lower band corresponds to ssDNA, whereas the upper band represents dsDNA. In samples where DNA-1 and DNA-2 were mixed, the intensity of the ssDNA band was diminished, and the dsDNA band appeared more prominent. These results confirm that 68-bp dsDNA was successfully formed through annealing, regardless of whether the DNA contained 5mC, 2′-OMe, or MP modifications. To evaluate the thermal stability of the chemically modified dsDNAs, melting temperature (*T*_m_) measurements were performed (Fig. S2). The *T*_m_ of the unmodified DNA duplex was 76 °C, whereas the *T*_m_ values of the modified dsDNAs were 77 °C for 5mC, 71 °C for 2′-OMe, and 72 °C for MP, respectively. These results suggest that all modified DNA duplexes are sufficiently stable under physiological conditions (37 °C). It is well established that 5mC is a modification that stabilizes DNA duplexes,^16^ and the observed increase in *T*_m_ relative to unmodified DNA is consistent with previous findings. By contrast, the 2′-OMe modification introduces a sugar structure similar to RNA, which possesses a hydroxyl group at the 2′ position. Because RNA duplexes are generally less stable than DNA duplexes, the decrease in *T*_m_ for the 2′-OMe-modified DNA is considered to reflect this property. To investigate the effect of chemical modifications on the helical structure of the DNA duplexes, circular dichroism (CD) spectroscopy was performed. The CD spectra of the modified DNA duplexes are shown in Fig. 2B. Notably, the DNA duplex modified with 2′-OMe exhibited a blue shift of the positive CD band peak from approximately 280 nm toward shorter wavelengths compared with the unmodified DNA duplex, consistent with a partial shift toward A-form character. In CD spectra of dsDNA, B-form DNA typically exhibits a positive peak in the 260–280 nm range, whereas A-form DNA shows a peak closer to 260 nm.^17^ Therefore, the spectral shift suggests that the 2′-OMe-modified dsDNA adopts a conformation more similar to that of A-form DNA than unmodified dsDNA. In addition, as indicated by the orange curve, the MP-modified DNA showed a smaller positive peak near 280 nm compared with the others, suggesting that it may adopt a relatively more dynamic or flexible duplex structure.

**Fig. 2:**
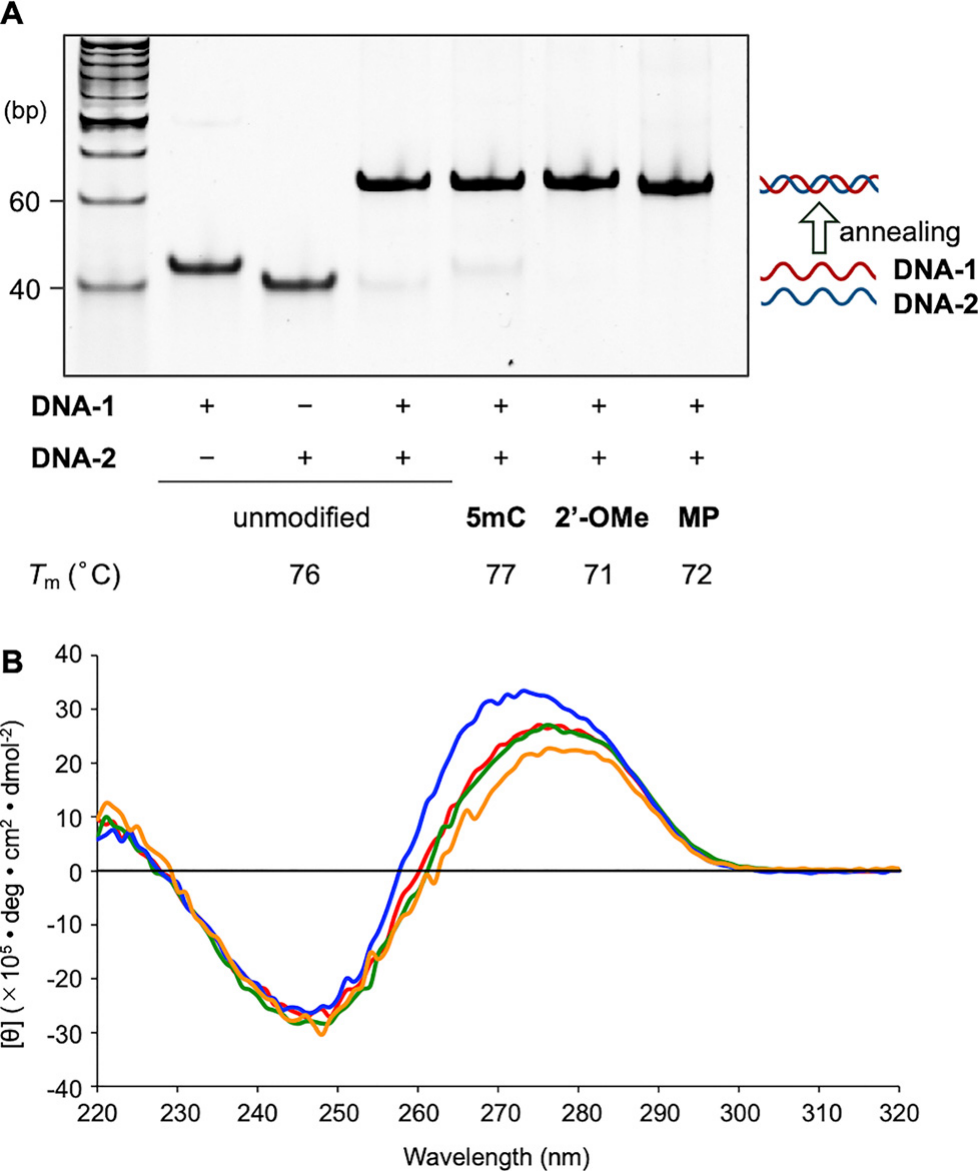
Effects of chemical modifications on DNA duplex stability and structure. (A) Sequences of the DNA strands used in this study. Chemically modified nucleotides are highlighted in red. (B) Thermal stability of chemically modified DNA duplexes. (C) Circular dichroism (CD) spectra of DNA duplexes bearing different chemical modifications. Red, unmodified; green, 5mC; blue, 2′-OMe; orange, MP.

To investigate the effect of chemical modifications on the binding affinity of dsDNA to cGAS, a gel shift assay was performed. DNA duplexes prepared by annealing were mixed with cGAS and incubated at 37 °C for 1 h before electrophoresis. In each of the four gel images, the lower band corresponds to DNA duplexes unbound to cGAS, whereas the upper band represents DNA duplexes bound to cGAS (Fig. 3A). In all cases, as the concentration of cGAS increased, the intensity of the lower band decreased, and the upper band became more prominent, indicating increased binding of dsDNA to cGAS. To assess the degree of cGAS binding quantitatively, the intensity of the lower bands was measured using ImageJ image analysis software. The vertical axis represents the relative amount of unbound DNA duplex (Fig. S3). A lower value indicates stronger binding to cGAS. DNA duplexes modified with 5mC or 2′-OMe showed similar binding affinity to that of unmodified DNA, whereas those with MP modification exhibited significantly reduced binding. The binding between cGAS and DNA involves electrostatic interactions between a groove formed by positively charged residues on cGAS and the negatively charged phosphate backbone of DNA (Fig. 1B).^14^ Because MP modification replaces one of the negatively charged oxygen atoms in the phosphate diester bond with a neutral methyl group, the reduced binding affinity observed for MP-modified DNA is likely due to the loss of this negative charge. To evaluate the ability of chemically modified DNA duplexes to activate cGAS, we quantified the signaling molecule cGAMP produced by activated cGAS. DNA duplexes prepared by annealing were incubated with cGAS at 37 °C for 1 h, followed by the addition of ATP and GTP and further incubation for 2 h to allow cGAMP synthesis. Quantification of cGAMP was performed using two methods; the first involved high-performance liquid chromatography (HPLC). Analysis of the reaction mixture by HPLC revealed peaks at approximately 13 min for GTP, 16.4 min for ATP, and 17.8 min for cGAMP. The amount of cGAMP produced was quantified by calculating the area under the cGAMP peak (Fig. S4). Compared with unmodified DNA duplexes, those modified with 5mC produced a smaller amount of cGAMP, whereas those with 2′-OMe modifications tended to produce higher amounts. By contrast, cGAMP was not detected in samples containing MP-modified DNA duplexes. The second quantification method utilized an ELISA kit designed for cGAMP detection (2′3′-Cyclic GAMP ELISA kit, Invitrogen). DNA duplexes containing 2′-OMe modifications significantly increased cGAMP production compared with unmodified dsDNA (Fig. 3B). Consistent with the HPLC results, cGAMP levels were barely detectable in samples containing MP-modified dsDNA. To elucidate how different chemical modifications affect cGAS activation, we conducted experiments using THP1-Lucia ISG cells. These cells are genetically engineered to express a secreted luciferase reporter gene under the control of an IRF3-inducible promoter, which is downstream of the cGAS–STING signaling pathway. Thus, luciferase activity serves as an indicator of cGAS activation (Fig. 3C). In this experiment, DNA duplexes were transfected into THP1-Lucia ISG cells using the Lipofectamine LTX Reagent, followed by a 24 h incubation. The amount of luciferase secreted into the culture supernatant was then quantified using the QUANTI-Luc 4 Lucia/Gaussia detection reagent. Consistent with the results of the *in vitro* cGAS activity assay, DNA modified with 2′-OMe induced higher cGAS activity compared with unmodified dsDNA in living cells. By contrast, MP-modified DNA did not activate cGAS sufficiently.

**Fig. 3:**
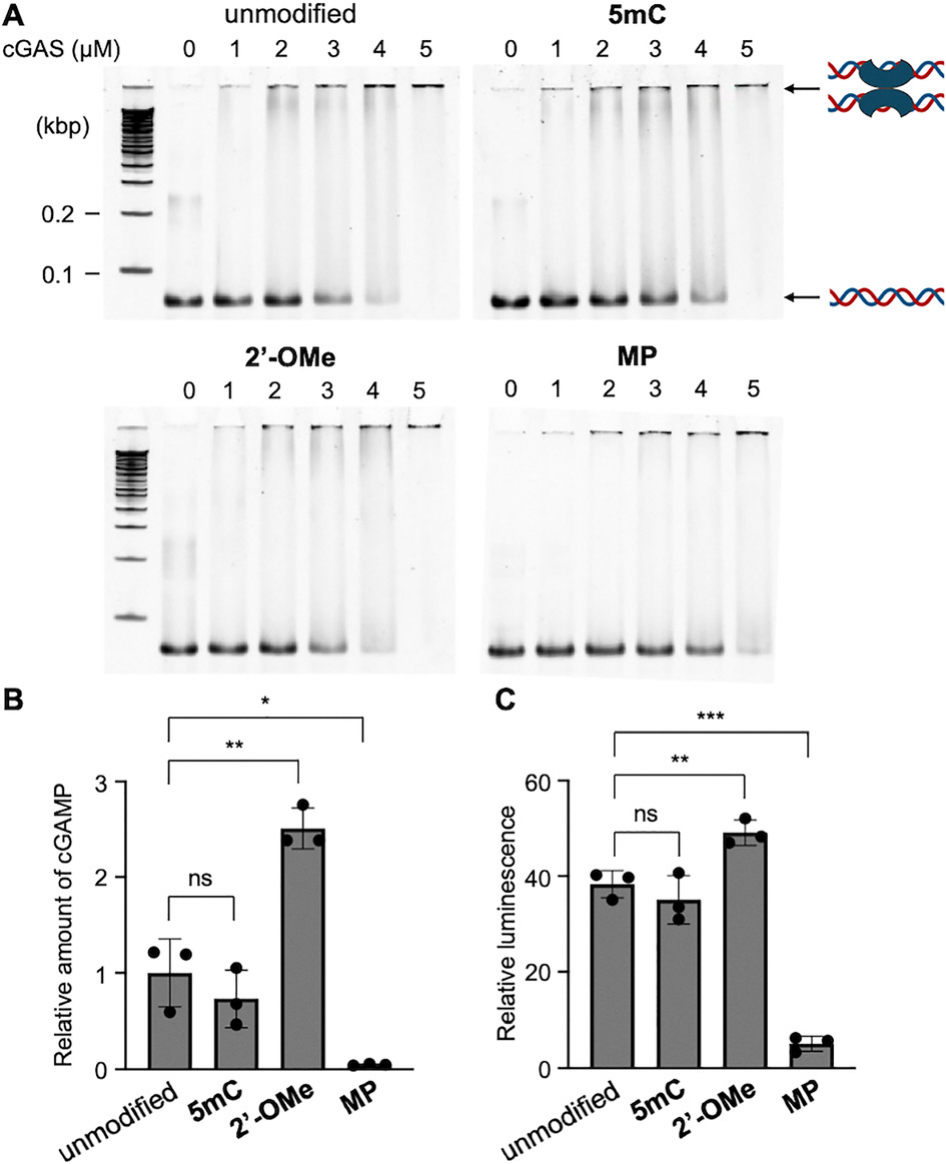
Effects of chemical modifications on cGAS activation by DNA duplexes. (A) Complex formation between cGAS and DNA duplexes. (B) Quantification of cGAMP production measured by ELISA. (C) Quantification of cGAMP production in living cells using a luciferase-based assay. Three independent experiments were averaged, and the error bars represent standard deviations. ns, not significant, *P < 0.05, **P < 0.01 and ***P < 0.001, unpaired Student *t* test.

To investigate phase separation, which is reported to be associated with cGAS activation, we observed condensate formation induced by cGAS–DNA complexes. DNA duplexes were stained with Hoechst dye by mixing them with cGAS and Hoechst 3342, and the resulting condensates were visualized using confocal microscopy (Fig. 4A). The condensate sizes were quantified using the ImageJ software (Fig. 4B). Although condensate formation with 2′-OMe-modified dsDNA was initially delayed (within the first 10 min after mixing with cGAS), large condensates were observed after 60 min. By contrast, MP-modified dsDNA did not result in noticeable condensate formation even after 60 min. These findings are consistent with the cGAS activity results shown in Fig. 4B and Fig. S4, confirming that phase separation of cGAS–dsDNA complexes plays a significant role in cGAS activation. To evaluate the properties of the observed condensates further, we assessed the fluidity of condensates formed by unmodified DNA and 2′-OMe-modified dsDNA using fluorescence recovery after photobleaching (FRAP). In this method, fluorescence is first quenched by intense excitation light, and the recovery of fluorescence over time is used to measure fluidity (Fig. 4C). For both types of dsDNA, fluorescence recovery slowed as the incubation time after mixing with cGAS increased, indicating a gradual loss of condensate fluidity (Fig. 4D). These findings suggest that cGAS and DNA molecules within the condensates are highly mobile and dynamically rearranged at early time points, but progressively lose mobility as the condensates undergo a liquid-to-solid transition, ultimately maturing into a gel-like state.^9^ In the case of 2′-OMe–modified dsDNA, condensate formation was too slow to allow FRAP measurements within the 3–10 min time window after mixing. The reduced thermal stability may reflect subtle changes in duplex dynamics and local flexibility, which could facilitate multivalent cGAS–DNA interactions and condensate rearrangement during LLPS. Although the precise contribution of duplex flexibility remains unclear, this possibility is consistent with the enhanced LLPS behavior observed for the 2′-OMe-modified duplex. While the present LLPS analysis is primarily qualitative, the observed condensate behavior was consistent with cGAS activation. More quantitative characterization of condensate properties will be important for future mechanistic studies.

**Fig. 4:**
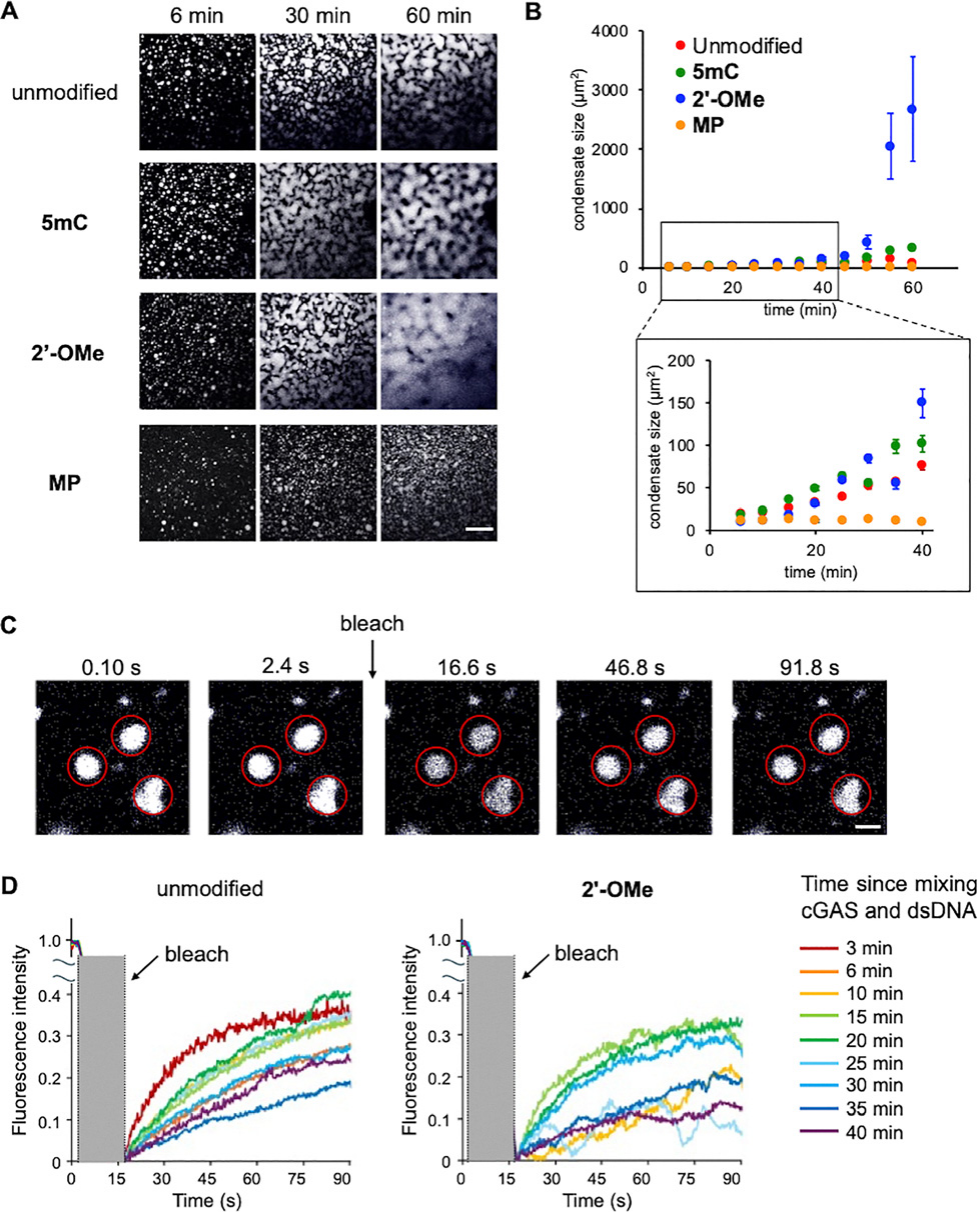
Effects of chemical modifications on LLPS of dsDNA duplexes with cGAS. (A) Time-lapse observation of condensate formation between chemically modified dsDNA duplexes and cGAS. Scale bars indicate 50 µm. (B) Changes in the size of condensates formed by chemically modified dsDNA duplexes and cGAS. Three independent experiments were averaged, and the error bars represent standard deviations. (C) Fluorescence photobleaching and recovery during time-lapse imaging. Red circles indicate the condensates. Scale bars indicate 5 µm. (D) Changes in fluorescence intensity of condensates after photobleaching at different mixing times of cGAS and dsDNA.

We investigated the effect of chemical modifications on the cytotoxicity of dsDNA using HeLa cells. dsDNA was transfected into cells using Lipofectamine LTX Reagent, and cell viability was measured 24 h later (Fig. 5A). In addition, the half-maximal inhibitory concentration (IC_50_) values were calculated (Fig. 5B). Compared with unmodified dsDNA, 5mC-modified dsDNA showed lower cytotoxicity, whereas 2′-OMe-modified dsDNA exhibited higher cytotoxicity. These results are consistent with the previously observed cGAS activity, suggesting that cytotoxicity induced by these two modifications is mediated through the cGAS–STING pathway. By contrast, MP-modified dsDNA exhibited a similar level of cytotoxicity to that of unmodified dsDNA. Given that MP-modified dsDNA does not activate cGAS, the observed cytotoxicity may be attributed to mechanisms independent of the cGAS–STING pathway. To explore the structural basis of cytotoxicity induced by MP modification, it is essential to consider that unmodified dsDNA carries a negative charge in its phosphodiester backbone. MP modification neutralizes this negative charge through methylation, which likely reduces the overall polarity of the molecule. This reduction in polarity may increase the likelihood of nonspecific interactions with cellular proteins, thereby increasing cytotoxicity. Moreover, previous studies have reported that MP-modified oligonucleotides are internalized through a cellular uptake pathway distinct from that of phosphodiester oligonucleotides,^18^ which may also contribute to the observed cytotoxicity independent of cGAS activation.

**Fig. 5:**
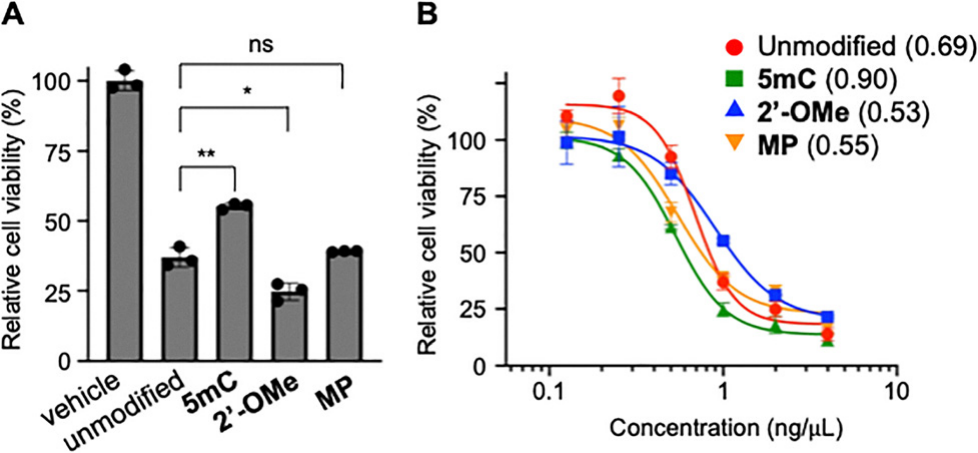
Cytotoxicity of chemically modified dsDNA. (A) Effects of chemically modified dsDNA (1 ng µL^−1^) on the viability of HeLa cells. (B) Dose–response curves for the viability of HeLa cells treated with chemically modified dsDNA. Numbers in parentheses indicate IC_50_ values (ng µL^−1^). Three independent experiments were averaged, and the error bars represent standard deviations. ns, not significant, *P < 0.05 and **P < 0.01, unpaired Student *t* test.

Among the examined DNA methylation modifications, 2′-OMe modification appears to be the most promising for enhancing innate immune activity. Condensates formed between cGAS and 2′-OMe-modified dsDNA potentiate activation of the cGAS–STING pathway; accordingly, 2′-OMe-modified dsDNA represents a promising immunotherapeutic agent or adjuvant for cancer therapy and viral vaccination. However, the positions and numbers of modification sites in the DNA duplexes used in this study were limited due to synthetic challenges. Therefore, the effects of each modification may not be generalizable and should be further investigated using other DNA duplex sequences and modification patterns. We recently developed several types of DNA or RNA hairpin pairs that exhibit cancer cell–selective cytotoxicity through multiple mechanisms,^15,19–21^ including, but not limited to, the cGAS–STING pathway, and the present study will further improve their anticancer activity.

RNA sequencing analysis revealed that both unmodified and 2′-OMe-modified dsDNA induced broad innate immune and inflammatory transcriptional programs, including enrichment of RIG-I-like receptor, NOD-like receptor, TNF, IL-17, and NF-κB signaling pathways (Fig. S5). Notably, the 2′-OMe-modified dsDNA exhibited stronger enrichment of multiple virus infection-related KEGG pathways, suggesting enhanced antiviral innate immune transcriptional signatures. These transcriptomic changes are consistent with the enhanced cGAS activation and cytotoxicity observed for the 2′-OMe-modified duplex. However, it remains unclear why 2′-OMe modification enhances condensate formation and enzymatic activity. Structural analysis of the complex between cGAS and the modified DNA duplexes by cryo-electron microscopy would be helpful for elucidating the underlying mechanism in future studies.

Collectively, our results support a unified mechanistic model in which chemical modifications of the DNA duplex modulate cGAS activation through changes in the physicochemical properties of the duplex. In particular, 2′-OMe modification appears to enhance multivalent cGAS–DNA interactions and condensate formation, possibly through subtle alterations in duplex conformation, hydration, and local dynamics. Enhanced LLPS formation is accompanied by increased cGAS enzymatic activity, broader innate immune transcriptional responses, and ultimately elevated cytotoxicity (Table 1). In contrast, MP modification suppresses cGAS activation despite retaining cytotoxicity, suggesting the involvement of cGAS-independent mechanisms. These findings collectively demonstrate that chemical modifications can differentially regulate both the structural and functional landscape of cGAS-mediated innate immune activation.

**Table 1:** Summary of the effects of 2′-OMe modification on cGAS-mediated innate immune activation

	Unmodified	2′-OMe
dsDNA properties	B-form	A-form-like tendency
LLPS formation	Smaller condensates	Larger condensates
cGAMP production	Moderate	Enhanced
Transcriptomic response	Induction of innate immune and inflammatory pathways	Stronger enrichment of antiviral and inflammatory transcriptional programs
Cytotoxicity	Moderate	Enhanced

## Conclusions

Modifications of the sugar, nucleobase, and phosphodiester linkage of dsDNA modulated the activity of the cGAS–STING pathway, with 2′-OMe-modified DNA exhibiting enhanced cGAMP production and cytotoxicity. We further demonstrated that the strong activation property of 2′-OMe-modified DNA depends on the efficient formation of condensates, which play a crucial role in establishing the reaction environment between DNA and cGAS. The present study highlights the importance of designing chemically modified dsDNA duplexes rationally for the development of immune-based anticancer drugs and vaccine adjuvants.

## Author contributions

K. M. and A. O. conceived and directed the study. M. B. and M. Y. performed the experiments and analyzed the data. All the authors prepared the manuscript.

## Conflicts of interest

There are no conflicts to declare.

## Supplementary Material

CB-007-D6CB00059B-s001

## Data Availability

All data are available in the manuscript or the accompanying supplementary information (SI). Supplementary information: includes the Materials and Methods section, quantitative data for cGAMP, and the results of the RNA-seq analysis. See DOI: https://doi.org/10.1039/d6cb00059b.
