# Supplementary material for: Methylation of DNA duplexes regulates cGAS-mediated innate immune activation via condensate formation
Source: RSC Chem Biol. 2026 Jun 4;7(7):1302–8. doi: 10.1039/d6cb00059b (PMC13250686; doi:10.1039/d6cb00059b)
Supplement: CB-007-D6CB00059B-s001 [file CB-007-D6CB00059B-s001.pdf]

Supplementary Information (SI) for RSC Chemical Biology.
This journal is © The Royal Society of Chemistry 2026

Supplementary Information

Methylation of DNA duplexes regulates cGAS-mediated innate immune activation via condensate formation

Kunihiko Morihiro,*[a] Manami Baba,[a] Moeko Yamada,[a] and Akimitsu Okamoto*[a]

[a] Department of Chemistry and Biotechnology, Graduate School of Engineering, The University of Tokyo, Bunkyo-ku, Tokyo 113-8656, Japan

*To whom correspondence should be addressed. Email: morihiro@chembio.t.u-tokyo.ac.jp (K.M.) or okamoto@chembio.t.u-tokyo.ac.jp (A.O.)

Table of Contents

	Material and methods	S2
**Fig. S1**	Illustration of the “ladder-like arrangement” of cGAS dimers on a long DNA duplex	S4
**Fig. S2**	Melting curves of DNA duplexes	S5
**Fig. S3**	Quantitative analysis of dsDNA unbound to cGAS	S6
**Fig. S4**	Quantification of cGAMP production by HPLC	S7
**Fig. S5**	RNA sequencing analysis	S8

Material and methods

General information. Oligonucleotides were purchased from Hokkaido System Science, GeneDesign, and ATDBio. THP1-Lucia ISG cells purchased from InvivoGen (thpl-isg). HeLa cells were obtained from the RIKEN BioResource Research Center (RCB No. 0007).

***T_m* measurement. DNA-1** and **DNA-2** (2 µM each) were annealed in TE buffer containing 50 mM NaCl and 1.25 mM $MgCl_2$. Melting temperature (T_m) measurements were performed using a spectrophotometer by heating the samples from 15 to 95 °C at a rate of 1 °C/min.

Gel shift assay. DNA-1 and **DNA-2** (1 µM each) were annealed in TE buffer containing 500 mM NaCl and 1.25 mM $MgCl_2$. After the addition of cGAS (0–5 µM) and incubation for 1 h at 37 °C, electrophoresis was performed on a 5% native PAGE gel at 150 V for 25 min at 4 °C. Band intensities were quantified using ImageJ software.

CD measurement. The CD spectra of the duplexes were recorded using a JASCO J-820 spectropolarimeter. **DNA-1** and **DNA-2** were dissolved in TE buffer containing 50 mM NaCl and 1.25 mM $MgCl_2$. Samples were heated to 100 °C and then slowly cooled to room temperature. Spectra were acquired from 210 to 320 nm at room temperature using a quartz cuvette. The scanning speed was set to 50 nm/min, and the response time was 1 sec. Mean residue ellipticity was calculated using the instrument's analytical software.

Quantification of cGAMP by HPLC. Annealed **DNA-1** and **DNA-2** (5 µM each), cGAS (10 µM), $MgCl_2$ (10 mM), and $ZnCl_2$ (100 µM) were mixed and incubated at 37 °C for 1 h. ATP (100 µM) and GTP (100 µM) were then added, followed by further incubation at 37 °C for 2 h. The reaction was quenched by heating at 95 °C for 5 min. Samples were filtered and analyzed by HPLC using an acetonitrile gradient from 0 to 10% over 30 min. Peaks eluting at 0–8 min and 30–40 min were excluded, and peaks with areas greater than 3000 µV·s and heights greater than 50 µV were extracted and integrated.

Quantification of cGAMP by ELISA. cGAMP was synthesized using the same procedure as described for HPLC-based quantification. ELISA measurements were performed using a 2',3'-cyclic GMP-AMP ELISA kit (Invitrogen) according to the manufacturer's instructions. Briefly, 50 µL of sample, 25 µL of cGAMP conjugate, and 25 µL of anti-cGAMP antibody were added to a 96-well strip plate coated with goat anti-rabbit IgG and incubated for 2 h at room temperature. After washing the plate, 100 µL of TMB substrate was added and incubated for 30 min at room temperature. The reaction was terminated by adding 50 µL of stop solution, and absorbance at 450 nm was measured using a microplate reader.

Cell culture. THP1-Lucia ISG cells were cultured at 37 °C under 5% CO_2 in RPMI-1640 medium supplemented with 10% fetal bovine serum (FBS), 1% penicillin–streptomycin solution, and 100 µg/mL Normocin. HeLa cells were cultured at 37 °C under 5% CO_2 in Dulbecco's modified Eagle's medium (DMEM) supplemented with 10% FBS and 1% penicillin–streptomycin solution.

cGAS activity assay in cells. THP1-Lucia ISG cells were seeded in a 96-well culture plate at a density of 1×10^5 cells per well and transfected with DNA–LTX complexes (1.0 µL LTX Reagent per 500 ng DNA and 1.0 µL PLUS Reagent per 1 µg DNA) using Opti-MEM as the transfection medium. Cells were cultured for 24 h, after which the cell suspension was centrifuged at 200 × g for 10 min. An aliquot of the supernatant (10 µL) was mixed with QUANTI-Luc 4 reagent (50 µL) in a 96-well white plate, and luminescence was measured with an integration time of 0.1 s.

Droplet imaging. Annealed dsDNA (10 µM) and cGAS (10 µM) were mixed in buffer containing 20 mM Tris–HCl, 150 mM NaCl, 1 mg/mL BSA, and 1 µL of Hoechst dye. Phase-separated droplets were imaged using a Nikon A1R+ confocal microscope equipped with a 40× objective. Droplet sizes were

quantified using ImageJ software. Fluorescence recovery after photobleaching (FRAP) experiments were performed on a Nikon A1R+ confocal microscope, and time-lapse images were acquired for 90 sec following photobleaching.

Cell viability assay. Cells were seeded in a 48-well culture plate at a density of 2.5×10^4 cells per well and cultured for 24 h. The culture medium was then removed, and cells were washed with PBS. DNA–LTX complexes (1.0 µL LTX Reagent per 500 ng DNA and 1.0 µL PLUS Reagent per 1 µg DNA) were prepared in Opti-MEM and transfected at a volume of 125 µL/well. Two hours after transfection, Opti-MEM was replaced with complete culture medium. Cells were further incubated for 24 h, after which PrestoBlue reagent was added (25 µL/well). Fluorescence was measured after 30 min with excitation at 555 nm and emission at 600 nm. Cells were transfected with dsDNA at 31.25, 62.5, 125, 250, 500, and 1000 ng per well, and IC_{50} values were calculated using a four-parameter logistic (4PL) model based on the six cell viability data points.

RNA sequencing. HeLa cells were passaged into a 24-well plate (500 µL per well, 5×10^4 cells per well) and grown to ≈80% confluence within 24 h. The medium was changed to Opti-MEM and the cells were transfected with dsDNA (105 ng) using Lipofectamine® LTX (2.1 µL). The medium was changed to a standard growth medium after 1 h, and the cells were incubated for 23 h. The cells were suspended, and RNA was extracted using RNAiso Plus (TaKaRa Bio). Sequencing libraries were generated using the TruSeq Stranded mRNA Kit (Illumina), according to the manufacturer's instructions. Sequencing was performed for 51 cycles using HiSeq Reagent Kit v2 on a HiSeq® 2500 Rapid instrument (Illumina). Base-calling was performed on the instrument and Fastq files were generated using CASAVA (Illumina). Fastq files of RNA-seq reads were mapped to the human transcriptome references. Fragments per kilobase of exon per million fragments mapped (FPKM) values were calculated for each UCSC gene.

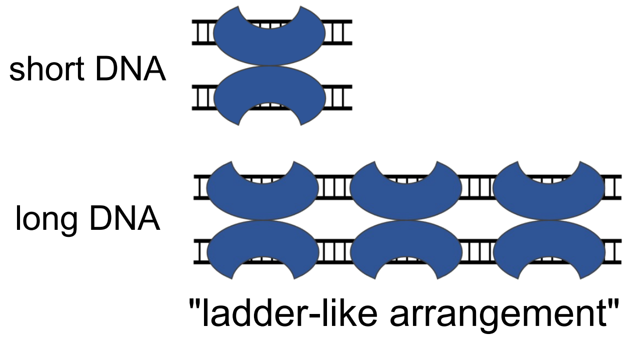

Fig. S1. Illustration of the “ladder-like arrangement” of cGAS dimers on a long DNA duplex.

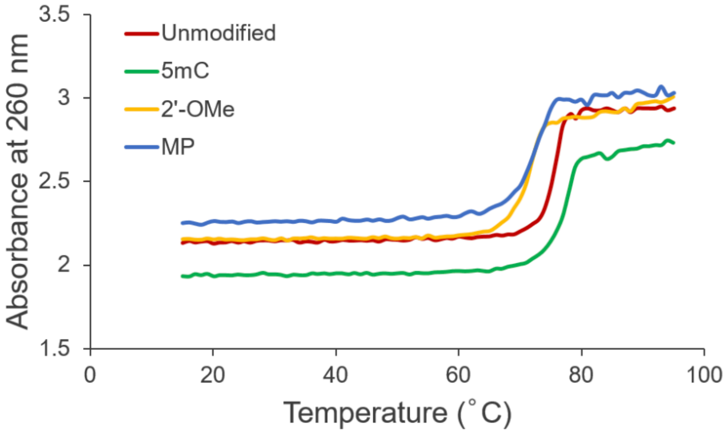

Fig. S2. Melting curves of DNA duplexes.

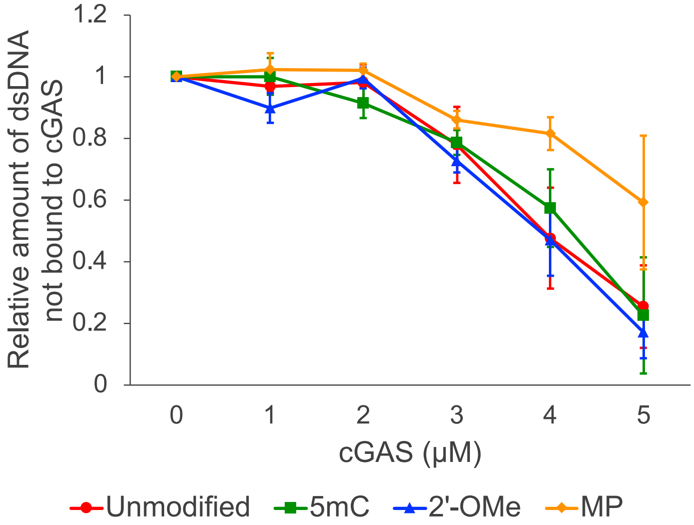

Fig. S3. Quantitative analysis of dsDNA unbound to cGAS. Three independent experiments were averaged, and the error bars represent standard deviations.

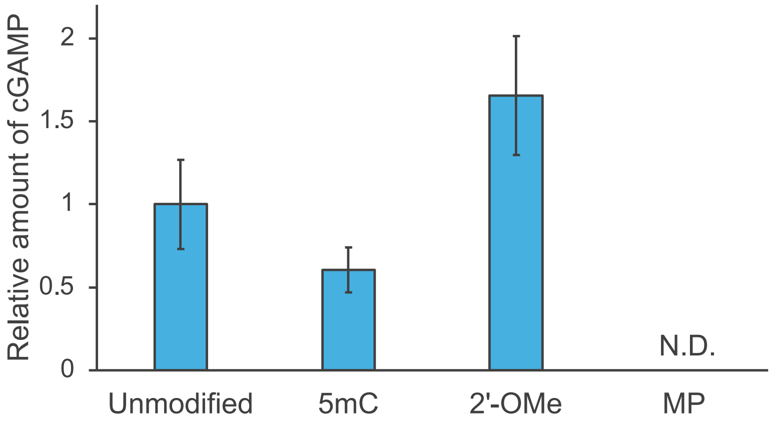

Fig. S4. Quantification of cGAMP production by HPLC. Three independent experiments were averaged, and the error bars represent standard deviations.

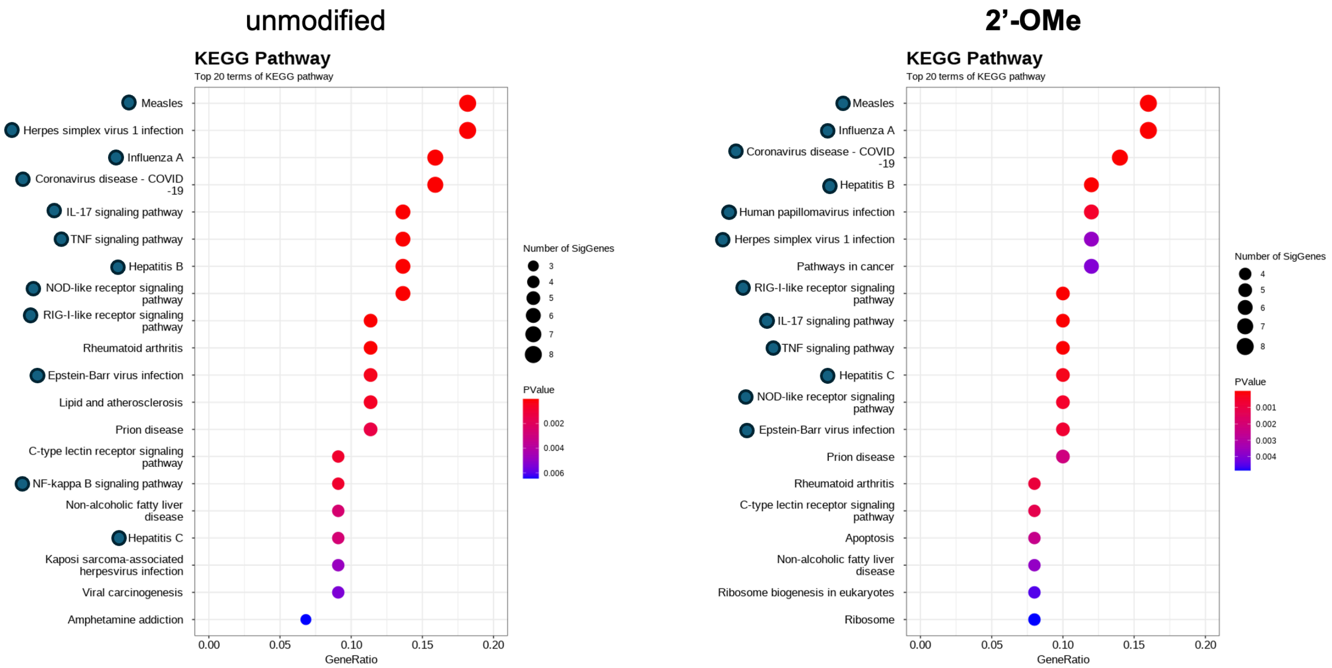

Fig. S5. RNA sequencing analysis. The blue circles are related to immunity or inflammation.
